# Genetic Characterization of the Exceptionally High Heat Resistance of the Non-toxic Surrogate *Clostridium sporogenes* PA 3679

**DOI:** 10.3389/fmicb.2017.00545

**Published:** 2017-04-03

**Authors:** Robert R. Butler, Kristin M. Schill, Yun Wang, Jean-François Pombert

**Affiliations:** ^1^Department of Biology, Illinois Institute of TechnologyChicago, IL, USA; ^2^United States Food and Drug Administration, Center for Food Safety and Applied NutritionBedford Park, IL, USA

**Keywords:** *Clostridium sporogenes*, *Clostridium botulinum*, PA 3679, *SpoVA*, dipicolinic acid, spore heat resistance, food sterilization, horizontal gene transfer

## Abstract

*Clostridium sporogenes* PA 3679 is a non-toxic endospore former that is widely used as a surrogate for *Clostridium botulinum* by the food processing industry to validate thermal processing strategies. PA 3679 produces spores of exceptionally high heat resistance without botulinum neurotoxins, permitting the use of PA 3679 in inoculated pack studies while ensuring the safety of food processing facilities. To identify genes associated with this heat resistance, the genomes of *C. sporogenes* PA 3679 isolates were compared to several other *C. sporogenes* strains. The most significant difference was the acquisition of a second *spoVA* operon, *spoVA2*, which is responsible for transport of dipicolinic acid into the spore core during sporulation. Interestingly, *spoVA2* was also found in some *C. botulinum* species which phylogenetically cluster with PA 3679. Most other *C. sporogenes* strains examined both lack the *spoVA2* locus and are phylogenetically distant within the group I *Clostridium*, adding to the understanding that *C. sporogenes* are dispersed *C. botulinum* strains which lack toxin genes. *C. sporogenes* strains are thus a very eclectic group, and few strains possess the characteristic heat resistance of PA 3679.

## Introduction

*Clostridium botulinum, Clostridium baratii*, and *Clostridium butyricum* species produce various types of botulinum neurotoxin (BoNT), the causative agent of the neuroparalytic botulism poisoning (Dodds and Hauschild, 1989; Collins and East, 1998; Rossetto et al., 2014). These species cluster into six groups defined by their metabolic and physiological traits (Collins and East, 1998; Rossetto et al., 2014). Group I (proteolytic) *C. botulinum* strains are particularly important to the food industry, as they produce endospores of high heat resistance that may survive inadequate thermal processing strategies and result in food spoilage and foodborne botulism (Townsend et al., 1938; Gross et al., 1946; Ingram and Robinson, 1951; Stumbo et al., 1975; Rossetto et al., 2014). *Clostridium sporogenes* is closely related to *C. botulinum* group I strains, but differs in two characteristic respects: it lacks the BoNT toxin genes and it produces spores with even higher heat resistance (Nakamura et al., 1977; Bull et al., 2009; Brown et al., 2012; Diao et al., 2014).

*C. sporogenes* PA 3679 (PA 3679) is widely used in testing commercial thermal food processing procedures for their ability to prevent foodborne botulism in shelf-stable products (McClung, 1937; Brown et al., 2012; Rossetto et al., 2014). PA 3679 is a non-toxic surrogate possessing higher heat resistant spores than group I *C. botulinum*, providing a safe alternative test organism that ensures neurotoxic spores have been eliminated during the thermal process without introducing the target pathogen to the food processing facilities (Brown et al., 2012; Diao et al., 2014). PA 3679 was originally isolated from spoiled canned corn in 1927 by E.J. Cameron of the National Canner's Association (Townsend et al., 1938; Brown et al., 2012). However, the properties of PA 3679 that give it such high heat resistance have not been well explored at a genetic level.

Genes associated with spore heat resistance in endospore formers focus on three properties: (1) DNA damage prevention (and repair) (2) dipicolinic acid (DPA) and cation concentrations in the spore core and (3) the spore's core water content (Setlow, 2006, 2007, 2014b). Genes of particular interest are those under control of the sporulation sigma factors σ^G^ and σ^F^ (forespore-specific) or σ^E^ and σ^K^ (mother cell-specific) (Eichenberger et al., 2003; Molle et al., 2003; Huang et al., 2004; Dürre, 2005; Wang et al., 2006).

High temperatures destabilize DNA and increase the incidence of depurination (Setlow, 2007, 2014b). In metabolically suspended cells, accumulated mutations cannot be repaired until germination resumes cellular activity (Setlow, 2006, 2007, 2014b). In virtually all reported endospore formers, the presence of α/β-type small acid-soluble spore proteins (SASP) prevent this damage by binding to and stabilizing DNA in its A-form orientation (Setlow, 2007; Lee et al., 2008). This binding mechanism is suggested to require two conserved domains: a germination protease (*gpr*) cleavage domain, and a DNA-binding domain that facilitates the DNA-SASP adduct (Cabrera-Martinez and Setlow, 1991; Setlow, 2007; Lee et al., 2008; Wetzel and Fischer, 2015). This α/β-type SASP DNA protection method is highly conserved and so effective that wild-type spores that are killed by wet heat exhibit minimally damaged DNA, suggesting the disruption of some other spore component (Setlow, 2007, 2014b).

The concentration of DPA in the spore core, chelated in a 1:1 ratio with divalent cations (often Ca^2+^), contributes to several spore functions (Granger et al., 2011; Setlow, 2014b). The magnitude of these effects can differ depending on the type of cation involved (Bach and Gilvarg, 1966; Paidhungat et al., 2000; Ragkousi et al., 2003; Setlow, 2014a,b). During sporulation, Ca^2+^-DPA creates a high acidity, low water environment in the spore core and binds remaining free water therein (Paidhungat et al., 2000; Setlow, 2006, 2014b; Paredes-Sabja et al., 2008b; Donnelly et al., 2016). This core dehydration aids in DNA-SASP association, and more importantly prevents damage to spore proteins essential for revival and germination (Setlow, 2006, 2014a,b; Paredes-Sabja et al., 2008b).

DPA is synthesized in the mother cell by shunting the product of DapA, dihydrodipicolinic acid (DHDPA), from the process of lysine biosynthesis (Daniel and Errington, 1993; Orsburn et al., 2010). The dicistronic *spoVF* operon codes for dipicolinic acid synthase subunits A and B (*dpaA/spoVFA* and *dpaB/spoVFB*), which convert DHDPA to DPA. Although the *spoVF* operon has been identified in many *Bacillus* species (Daniel and Errington, 1993; Onyenwoke et al., 2004) and *Peptoclostridium difficile* (Donnelly et al., 2016), this operon is not found in all endospore formers. Other members of the class Clostridia, including *Clostridium perfringens* and *Thermoanaerobacter* spp., lack *spoVF* (Onyenwoke et al., 2004). In *C. perfringens*, an alternate dipicolinic acid synthase, EtfA, has been demonstrated to produce DPA *in vitro* and *in vivo*, with knockout mutants lacking this metabolite (Orsburn et al., 2010). Following synthesis, DPA is transported to the core by three to seven products coded by the *spoVA* operon (Tovar-Rojo et al., 2002; Paredes-Sabja et al., 2008b; Li et al., 2012; Perez-Valdespino et al., 2014). Of these, products coded by *spoVAC, spoVAD* and *spoVAE* seem particularly important and are especially well conserved in both *Bacillus* and *Clostridium* species (Onyenwoke et al., 2004; Paredes-Sabja et al., 2008b; Donnelly et al., 2016).

In addition to DPA, several other genes are associated with core dehydration, though their roles are less clear. Spore maturation proteins A and B (products of *spmA* and *spmB*) both play a significant role in reducing core water content, though the mechanism is not understood (Paredes-Sabja et al., 2008a; Orsburn et al., 2009). The *dac* genes (*dacA, dacB, dacC*, and *dacF* in *B. subtilis*) code for D-alanyl-D-alanine carboxypeptidases which regulate peptidoglycan crosslinking. Both *dacB* and *dacF* genes are under the control of sporulation specific sigma factors, and their products regulate spore cortex formation (Popham et al., 1999). Knockout mutants lacking either gene show diminished heat resistance, presumably due to reduced cortex integrity under high heat conditions (Popham et al., 1999; Paredes-Sabja et al., 2008a; Orsburn et al., 2009).

In a previous study, we sequenced eight *C. sporogenes* samples labeled “PA 3679” obtained from a variety of sources and which displayed differential heat resistance (Schill et al., 2016). From our analyses, we distinguished two distinct clades of *C. sporogenes* isolates. Clade I isolates had significantly lowered heat resistance, with two (1990 and 2007) featuring near-identical genotypes. Clade I isolates did not survive heat treatment at 105°C for 5 min and displayed D_97°C_ and D_100°C_ values of 2.97 and 2.28 min, respectively (the decimal reduction time, D, is equal to the time required under a given condition to destroy a population of microorganisms by one logarithm). In contrast, all isolates from clade II exhibited near-identical genotypes and heat resistance profiles of the original PA 3679 isolate by E.J. Cameron, with an estimated D_121°C_ of 1.28 min (Diao et al., 2014), and survived thermal processing at temperatures from 117°C to 121°C. Given the two clades of *C. sporogenes* with differing heat resistance, we were presented with an opportunity to elucidate the specific genomic differences conferring the exceptional heat tolerance of PA 3679 spores.

## Materials and methods

### Genomes used in study

Eight genomes used in this study (Table 1) were from our previous study (Schill et al., 2016). The annotations of *C. sporogenes* 1961-2 (LLZW02), *C. sporogenes* 2007 (LLES02), *C. sporogenes* 1990 (LLZV01), *C. sporogenes* PA 3679 1961-4 (LLZT01), *C. sporogenes* PA 3679 Camp (LKKY02), *C. sporogenes* PA 3679 FDA (LJTA01), and *C. sporogenes* PA 3679 NFL (LJSZ01) and *C. sporogenes* PA 3679 UW (LFVV01) were updated to reflect the information from this study.

**Table 1:** ***Clostridium sporogenes* isolates used in Schill et al. (2016)**.

**Short name**	**Full name**	**Spore heat resistance group**	**Source**
1961-2	*Clostridium sporogenes* 1961-2	clade I (low heat)	Contaminant of ATCC 7955 NCA3679
1990	*Clostridium sporogenes* 1990	clade I (low heat)	Contaminant of ATCC 7955 NCA3679
2007	*Clostridium sporogenes* 2007	clade I (low heat)	Contaminant of ATCC 7955 NCA3679
1961-4	*Clostridium sporogenes* PA 3679 1961-4	clade II (high heat)	ATCC 7955 NCA3679
Camp	*Clostridium sporogenes* PA 3679 Camp	clade II (high heat)	Campbell's Soup Company
FDA	*Clostridium sporogenes* PA 3679 FDA	clade II (high heat)	U.S. Food and Drug Administration
NFL	*Clostridium sporogenes* PA 3679 NFL	clade II (high heat)	National Food Laboratory
UW	*Clostridium sporogenes* PA 3679 UW	clade II (high heat)	Johnson Lab, University of Wisconsin-Madison

### Pan-genomic analysis

For pan-genomic comparison, the eight strains previously described were clustered using Roary 3.6.2 (Page et al., 2015) using a 70% identity threshold. Roary's core gene alignment was trimmed using BMGE 1-1 (Criscuolo and Gribaldo, 2010) to 2,511,737 sites across 2,751 core genes. PhyML 3.1 (Guindon et al., 2010) was used with GTR + I + F + G (4 categories) to generate a maximum likelihood (ML) tree for clustering. Roary_plots.py (https://github.com/sanger-pathogens/Roary/tree/master/contrib/roary_plots) was used to generate the orthologous cluster map. Orthologs unique to the five clade II isolates were examined and those related to sporulation were investigated.

Roary was used with 23 *C. sporogenes* (including the eight in this study) and 15 group I *C. botulinum* genomes, to generate a concatenated nucleotide alignment of 389 core genes (216,294 sites) using a 70% identity threshold. A maximum likelihood tree was generated as above, with the addition of 100 bootstraps in PhyML.

To calculate pairwise mutational distances between the 38 group I *Clostridium*, Mash (Ondov et al., 2016) pairwise comparisons were plotted using metric multidimensional scaling with the cmdscale and igraph (Csárdi and Nepusz, 2006) packages implemented in R (R Core Team, 2016), using custom Perl scripts available via the Pombert Lab github page (https://github.com/PombertLab).

### Analysis of *spoVA* and conserved genes

Orthologous groups identified from the pan-genomic analysis were searched using known genes related to spore heat resistance. Additional homology searches using BLAST (Altschul et al., 1990) looked for any missed homologs. Both blastp and tblastn searches were conducted using known reference genes (See Supplementary Table 1). Orthologous groups for each gene were compared and aligned using Geneious 9.1.5 (Kearse et al., 2012). Conserved domains in the aligned clusters were revealed with InterProScan 5 (Jones et al., 2014).

### Analysis of the *spoVA* operon

Using the 38 *Clostridium* above, plus *C. tetani* E88, OrthoFinder 0.2.8 (Emms and Kelly, 2015) identified 6,168 orthologous groups, 840 of which were unique orthologs present in all 39 strains. All *spoVA* genes identified by the pan-genomic analysis were located in the ortholog groups produced by OrthoFinder. Neighboring genes and operons were also identified in all 38 group I *Clostridium* species examined. *spoVA2* operons and neighboring genes for several representative species were aligned and compared using EasyFig 2.2.2 (Sullivan et al., 2011). Conserved domains were identified using InterProScan 5, and predicted protein structures were calculated using the RaptorX webserver (Källberg et al., 2012).

## Results

### Pan-genome analysis

**Figure 1:**
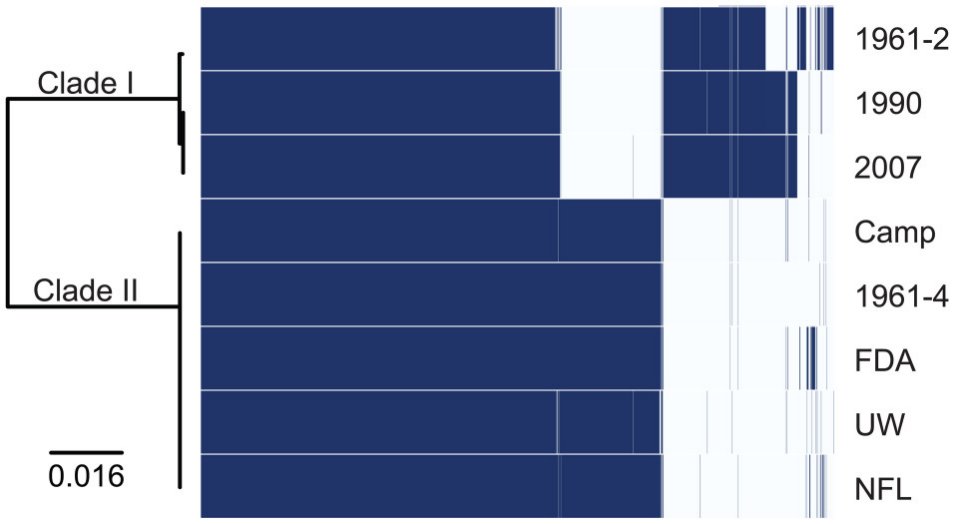
**Pangenomic cluster matrix of *Clostridium sporogenes* isolates**. A total of 4,899 orthologous clusters were identified; orthologs present or absent are indicated in blue or white, respectively. The maximum likelihood tree on the left was generated from an alignment of 2,751 core orthologs using a GTR + I + F + G model.

The Roary pan genome generated using the eight clade I and clade II *C. sporogenes* isolates contained a total of 4,899 distinct orthologous groups, 2,751 of which represented the core genes, each with a unique ortholog in all eight isolates (Figure 1). These core orthologs included many genes previously identified as related to spore heat resistance. The heat resistance core orthologs are further characterized in Supplementary Figure 1, and described later in detail. There were 751 ortholog groups that were present in the five clade II isolates, but absent in clade I. Of those, 278 ortholog groups code for hypothetical proteins. Seven of the 751 were sporulation specific, of which four ortholog groups were related to germination. The remaining three ortholog groups constituted a second set of *spoVA* genes not found in the clade I isolates, henceforth dubbed the *spoVA2* locus.

### Examination of the *spoVA2* operon

**Figure 2:**
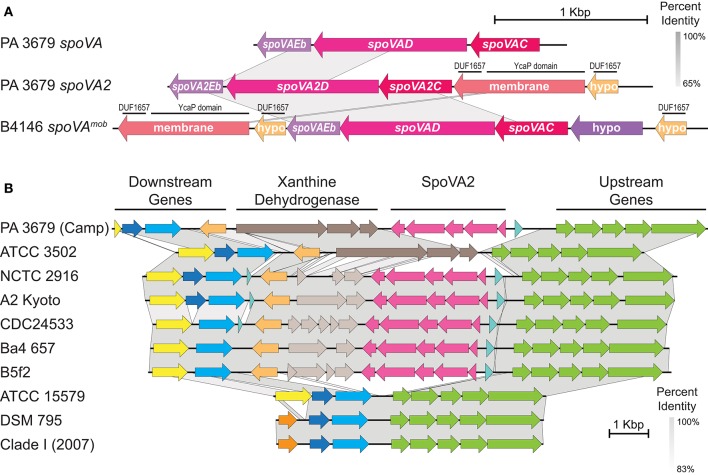
**Characteristic stage V sporulation A operon structures. (A)** The *spoVA* and *spoVA2* operons depicted are from *C. sporogenes* PA 3679 Camp; the *spoVA*^*mob*^ mobile element contained in a Tn*1546* transposon is from *Bacillus subtilis* B4146 (Berendsen et al., 2016). Pairwise blastn identity percentages are colored per a gray gradient (see legend in upper right). Gene names and conserved domains are displayed on the corresponding loci. Hypo, hypothetical protein; membrane, membrane protein; DUF, domain of unknown function. **(B)** Genomic neighborhood of the *spoVA2* operon. Pairwise blastn identity percentages are colored per a gray gradient (see legend in lower right). Gene/pseudogene clusters are broadly defined at the top. Genes encode the following proteins: Dark orange & yellow, GNAT acetyltransferases; Dark blue, riboflavin biosynthesis protein RibD; Light blue, DNA polymerase III epsilon/RNAse T family protein; Light orange, isochorismate hydrolase; Dark brown (left to right), putative xanthine dehydrogenase subunits XdhB, XdhA, and XdhC; Light brown, degenerate pseudogenes and partial CDS fragments with high homology to the xanthine dehydrogenase operon; Fuscia (left to right), SpoVAEb, SpoVAD, SpoVAC, membrane protein, and hypothetical protein; Aqua, hypothetical protein; Green (left to right), hypothetical protein, cytochrome C biogenesis protein ResB, thiol-disulfide oxidoreductase ResA, signal transduction response regulator, signal transduction histidine kinase.

As mentioned in the pan genomic analysis, a second locus of *spoVA* genes was found in a single pentacistronic operon, *spoVA2* (Figure 2A). InterProScan searches of the *spoVA2* genes revealed conserved domains from two types of *spoVA* operons. To explore this further, a collection of the *C. sporogenes* genomes in GenBank (at the time of writing) plus fifteen commonly studied group I *C. botulinum* strains and one *C. tetani* strain (Table 2) were clustered using OrthoFinder. For all five clade II isolates and five additional *C. botulinum* species, this *spoVA2* operon was conserved and clustered separately from the traditional *spoVA* operon, which was found in all 39 species. All 39 species showed similar *spoVA* loci as clustered in Orthofinder. The 38 group I Clostridia showed a conserved genomic neighborhood around the site of the *spoVA2* operon inclusion (Figure 2B). The *spoVA2* operon and its neighboring regions were perfectly conserved in all clade II (PA 3679) isolates, so only one representative sequence (Camp) is depicted in the figure. The clade I isolates similarly only had a single nucleotide difference across the whole region which didn't affect gene coding, so 2007 was chosen as the representative in Figure 2B.

**Table 2:** **Group I *Clostridia* used in *spoVA2* comparisons**.

**Strain name**	**Species**	**Toxin type**	**Accession**
AM1195	*Clostridium botulinum*	B6	LFPH01
ATCC 19397	*Clostridium botulinum*	A1	CP000726.1
ATCC 3502	*Clostridium botulinum*	A1	AM412317.1
B5f2	*Clostridium botulinum*	B5f2	ABDP01
Ba4 657	*Clostridium botulinum*	B5a4	CP001083.1
F230613	*Clostridium botulinum*	F1	CP002011.1
H04402-065	*Clostridium botulinum*	A5	FR773526.1
Hall	*Clostridium botulinum*	A1	CP000727.1
Kyoto	*Clostridium botulinum*	A2	CP001581.1
Langeland	*Clostridium botulinum*	F1	CP000728.1
Loch Maree	*Clostridium botulinum*	A3	CP000962.1
NCTC 2916	*Clostridium botulinum*	A1(B)	ABDO02
Okra	*Clostridium botulinum*	B1	CP000939.1
Osaka05	*Clostridium botulinum*	B6	BAUF01
Prevot 594	*Clostridium botulinum*	B	CP006902.1
11579	*Clostridium sporogenes*	–	JZJN01
66_CBOT	*Clostridium sporogenes*	–	JUYE01
85-3852	*Clostridium sporogenes*	–	JZJO01
87-0535	*Clostridium sporogenes*	–	JZJP01
88-0163	*Clostridium sporogenes*	–	JZJQ01
8-O	*Clostridium sporogenes*	–	LUAU01
ATCC 15579	*Clostridium sporogenes*	–	ABKW02
ATCC 19404	*Clostridium sporogenes*	–	LFPM01
Bradbury	*Clostridium sporogenes*	–	AGAH01
CDC23284	*Clostridium sporogenes*	–	LAGF01
CDC24533	*Clostridium sporogenes*	–	LAGH01
DSM 795^†^	*Clostridium sporogenes*	–	CP011663.1
DSM 795	*Clostridium sporogenes*	–	JFBQ01
NCIMB 10696	*Clostridium sporogenes*	–	CP009225.1
UC9000	*Clostridium sporogenes*	–	LJFK01
E88^a^	*Clostridium tetani*	–	AE015927.1

a *Not a group I Clostridium species, outgroup used for clustering and phylogeny*.

**Figure 3:**
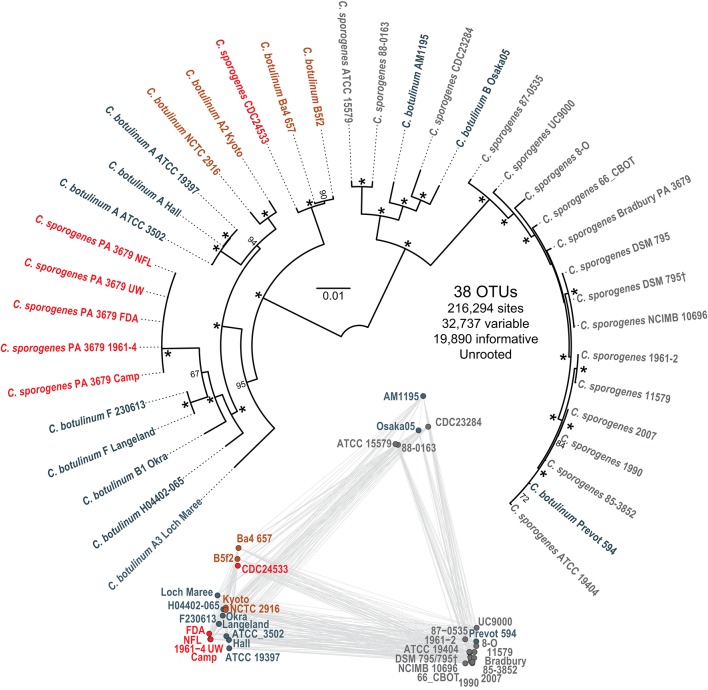
**Relationships between group I *Clostridium* strains**. *C. sporogenes* and *C. botulinum* strains containing the *spoVA2* operon are colored red and orange, respectively, whereas *spoVA2*-less *C. sporogenes* and *C. botulinum* strains are colored in gray and blue-gray, respectively. (Upper) Maximum likelihood tree computed using a GTR + I + G + F model and 100 bootstraps from a concatenated alignment of 389 core orthologous clusters. Bootstrap values >60 are shown at their respective nodes, with nodes present in all bootstrap replicates indicated by asterisks. (Lower) Metric multidimensional plot of pairwise genetic distances between whole *Clostridium* genomes.

† *Two distinct whole genome submissions from different groups for strain DSM 795 are available in GenBank. The cross identifies which strain corresponds to which accession number in Figure 3*.

The *spoVA2* operon itself was well conserved in all strains it was found in. In addition to SpoVAC, SpoVAD, and SpoVAEb, the operon encodes two other proteins: a hypothetical protein and a membrane protein (Figure 2A). Neither protein has domain similarity or sequence homology to SpoVAA or SpoVAB. Both feature a domain of unknown function (DUF), DUF1657 (IPR012452; PF07870), and the membrane protein contains an additional uncharacterized YcaP domain (PTHR34582) composed of three transmembrane domains and DUF421. Predicted 3D structures of the DUF1657 hypothetical protein, the YcaP/DUF1657 membrane protein and SpoVAC, SpoVAD, and SpoVAE are depicted in Supplementary Figure 2, with high similarity to previously reported examples. Also of note is the downstream neighbor of *spoVA2*, the xanthine dehydrogenase (*xdh*) operon. The *xdh* operon and a gene encoding isochorismate hydrolase are present in all *spoVA2* containing strains, as well as closely related *C. botulinum* A strain ATCC 3502 (which lacks *spoVA2*; Figure 2B). However, in several strains, the *xdh* genes are partial or pseudogenes.

### Characterization of SASP

A total of eight different SASP-encoding ortholog groups were found, each group containing an ortholog from every one of the eight investigated genomes. The traditional α/β-type SASP, with both a *gpr* cleavage domain (IPR018126; Prosite PS00304) and a DNA-binding domain (IPR018126; Prosite PS00684), was encoded by three of these orthologous groups. Translations of the genes in those groups, named *ssp1, ssp2*, and *ssp3*, also displayed the characteristic α/β-type SASP Pfam domain (IPR001448; PF00269).

A fourth SASP-encoding ortholog group showed high sequence conservation to a previously described *ssp4* in *C. perfringens* (Li and McClane, 2008; Li et al., 2009). The product coded by these *ssp4* orthologs had the characteristic α/β-type SASP Pfam domain, and the *gpr* cleavage domain, but lacked the conserved DNA-binding domain. The fifth SASP-coding ortholog group contained orthologs labeled *ssp5*. Again, translations displayed the conserved SASP Pfam domain, however it lacked both the *gpr* cleavage domain and the DNA-binding domain typical of α/β-type SASP.

The remaining three SASP-encoding ortholog groups exhibited the conserved domains and sequence similarity to minor types of SASP not associated with high heat resistance in previous studies: the H-type SASP and the *tlp* type SASP (Cabrera-Hernandez et al., 1999; Wetzel and Fischer, 2015). All of the SASP-encoding ortholog groups in this study appear to be monocistronic, and the amino acid alignments of each SASP is available in Supplementary Figure 1.

### Characterization of conserved sporulation genes

A number of additional sporulation-related orthologous groups were found with representative orthologs from all eight isolates. Six D-alanyl-D-alanine carboxypeptidase encoding orthologous groups were found, and their respective orthologous genes were dubbed *dac1* through *dac6*. One orthologous group, *dac4*, encoded proteins with high homology to DacF (blastp e-values above 1e-105) and contained the two expected conserved domains: Peptidase S11, N-terminal domain (IPR001967; Pfam PF00768) and Penicillin Binding Protein 5, C-terminal domain (PBP5_C) (IPR012907; Pfam PF07943). Two orthologous groups—*dac2* and *dac5*—encoded proteins similar to DacB (with blastp e-values above 1e-49) which characteristically have the same two conserved domains as DacF. The three remaining *dac* ortholog groups (*dac1, dac3*, and *dac6*) showed poor similarity to *dacB* or *dacF* and are likely D-alanyl-D-alanine carboxypeptidases unrelated to sporulation. The amino acid alignments of all Dac proteins are available in Supplementary Figure 1.

The *spoVF* operon, coding for DPA synthase subunits A and B, was not found in any of the eight isolates. Instead, three orthologous groups—containing orthologs dubbed *etfA_1, etfA_2*, and *etfA_3*—encoded products with high protein sequence similarity (blastp e-values above 1e-130) to EtfA from *C. perfringens*, an alternate DPA synthase. All three EtfA homologs were present in all eight genomes. Only EtfA_1 contained the correct array of conserved domains associated with *C. perfringens* EtfA. EtfA_3 lacked a Prosite conserved motif (IPR018206; PS00696) and EtfA_2 contained an extra conserved domain: N-terminal 4Fe-4S ferredoxin-type iron-sulfur binding domain (FerB) (IPR017896; Pfam PF00037). The amino acid alignments of the EtfA proteins are available in Supplementary Figure 1.

The orthologous groups for 4-hydroxy-tetrahydrodipicolinate synthase (DapA) and 4-hydroxy-tetrahydrodipicolinate reductase (DapB) were both present in all eight isolates, as was a second DapB orthologous group (encoded by *dapB_2*). Orthologous groups encoding spore maturation protein A (SpmA) and B (SpmB) were also found and contained an ortholog in all eight isolates. Other orthologs typically associated with germination were also identified in the isolates. Germination protease (*gpr*), putative germination protease (*yyaC*) and spore photoproduct lyase (*splB*) orthologs were also found in all eight genomes. Supplementary Table 1 summarizes the orthologous genes and includes those which were found in *C. botulinum* A strain ATCC 3502. Locus tags and further information for all the genes in this study can be found in Supplementary Table 1.

### Phylogenomic comparison

The phylogeny in Figure 3 (upper) depicts a branching of *C. sporogenes* and *C. botulinum* strains into two mixed groups. The majority of *C. sporogenes* strains are grouped together in the right group, though clade II (PA 3679) isolates group on the left. The majority of *C. botulinum* strains are in the left group, though several are present in the right group. All strains possessing the *spoVA2* locus are in the left group. The xanthine dehydrogenase operon and isochorismate hydrolase are present in all members of the left group, though degenerated in some strains, and absent in all strains in the right group. The clade II isolates form an extremely well conserved group consistent with coming from the same original spore crop. The clade I isolates also group as expected, showing similarity to several *C. sporogenes* strains and one *C. botulinum* strain, Prevot 594. The pairwise genetic distances comparison in Figure 3 (lower) shows consistent results with the core gene phylogenetic tree, however the species in the right branch of the phylogeny are split into two more distinct groups than the phylogenetic tree alone would suggest.

## Discussion

Ensuring the quality and safety of packaged foods is an ongoing process that is ideally unnoticed by the consumer when everything works as intended. While current methods for food preservation have an excellent track record, bacteria do evolve over time and there is a non-negligible risk that these methods may no longer be adequate in the near future (for example, the rise of antibiotic resistance is a sharp reminder that things can change quickly in the microbial world). Here, we were offered the opportunity to examine the genetic differences behind the low and high heat resistance of clade I and II isolates of *C. sporogenes*; a knowledge that could be applied to the detection and prevention of heat resistance in pathogenic species of *Clostridium* and other common foodborne pathogens. Importantly, we discovered that the genetic locus that most likely conveys a meaningful improvement in PA 3679 spore heat resistance is part of the bacterial mobilome, and that this heat resistance-conferring island could be, and likely has been, transferred from/to a number of pathogenic species. Our study also serves as a reminder that not all *C. sporogenes* isolates identified as PA 3679 strains actually possess the capability for high heat resistance and therefore cannot fulfill the role of non-toxic, thermal surrogates. Processes vetted using these deficient strains may not perform up to the desired specifications, with potentially dire implications for the safety of the foods packaged by these processes.

### Impact of the *spoVA2* locus

Undoubtedly, the most significant difference found between the low and high heat resistance isolates was the presence of a second set of *spoVA* genes in the clade II (PA 3679) group. This is not the first reported incidence of multiple *spoVA* operons in an endospore former. At least one group IV *Clostridium* species (Brunt et al., 2016), several species of *Geobacillus* and *Bacillus cereus*, as well as several of the more heat resistant *Bacilli* have multiple *spoVA* loci. Adding additional copies of *spoVA* in a mobile Tn*1546* transposon (*spoVA*^*mob*^, Figure 2A) creates an additive resistance effect, greatly increasing the concentration of DPA in the *Bacillus subtilis* spore core (a D_112.5°C_ increase from 0.2 min to 25.6 min with three copies; Berendsen et al., 2016). Our results are compatible with these previous observations, and lend to a compelling hypothesis about spore heat resistance. As spore formation is temporally limited, the SpoVA apparatus encounters a flow rate challenge. Increasing the flow rate with extra pumps can either move more DPA in the given time, or overcome losses due to diffusion (or both) resulting in a much higher concentration of DPA in the spore core. This effect should scale until the point that the maximal amount of DPA has been added, which has apparently not been reached in *Bacillus*, and our findings suggest the same for Clostridia, with the implication that further multiplication of this operon in the genetic paraphernalia of an endospore former may imbue it with the ability to survive current canning processes.

The origin of the PA 3679 *spoVA2* operon, however, is not entirely clear. This operon was not contained in the same Tn*1546* mobile element as in *Bacillus* species, but individual genes within the *spoVA2* locus—hypothetical protein, membrane protein, *spoVAC, spoVAD*, and *spoVAEb*—showed a higher sequence similarity to foreign loci than to the native *spoVA* locus in PA 3679 (Figure 2A). Blastn searches of the contiguous *spoVA2* operon gave high sequence homology (>80% identity, >99% query coverage) to the expected *C. botulinum* species from this study, plus *C. argentinese* CDC 2741, *C. neonatale, C. saccharobutylicum* DSM 13864, and *C. saccharoperbutylacetonium* N1-4. Given this information, horizontal acquisition seems more likely than a paralogous duplication event. This idea is furthered when considering the two additional genes (coding for the DUF1657 domain-containing and YcaP domain-containing proteins) which show a homology to *spoVA2*^*mob*^ from *B. subtilis* yet are absent in the native *spoVA* locus. The YcaP domain-containing protein is of particular interest as Berendsen et al. (2016) knocked out the orthologous protein in *spoVA*^*mob*^, which severely diminished spore heat resistance in *B. subtilis*. While the roles of SpoVAD (Li et al., 2012) and SpoVAC (Velásquez et al., 2014) are partially established in DPA transport, a full understanding of the roles of all *spoVA2* proteins needs further study.

The presence of additional *spoVA* operons in *Clostridium* species has not been previously explored, though it is a phenomenon that occurs not just in PA 3679, but also in several closely related *C. botulinum* species (Figure 2B) and at least one *C. argentinese* strain (Brunt et al., 2016). This might seem paradoxical as *C. botulinum* is generally considered to have lower spore heat resistance than *C. sporogenes*. However, (1) there is a large amount of variance and inconsistency in heat resistance data, owing to a variety of environmental factors involved, (2) the heat resistance of the *C. botulinum* species possessing the *spoVA2* locus has not been widely studied and (3) this study only examined a comparison of *C. sporogenes* strains. Perhaps *C. botulinum* species containing the *spoVA2* locus also feature increased heat resistance. This would present a considerable challenge designing thermal processing strategies which effectively eliminate this dangerous pathogen in a food product. Future studies will be required to explore other spore heat resistance factors that may differ between the *C. botulinum* and *C. sporogenes*.

### High conservation of SASPs

The α/β-type SASP have been recognized primarily for their function in maintaining spore DNA integrity when exposed to a variety of factors (Setlow, 2014b). Many studies of α/β-type SASP knockouts have demonstrated a significant loss of heat resistance when lacking a functional DNA protection mechanism (Setlow, 2007, 2014b). However, the protection provided by SASP is not additive, as only one or two of the paralogous SASP-encoding genes are expressed in large amounts, and confer maximal heat resistance (Setlow, 2014b). The presence of eight SASP-encoding orthologous groups in our isolates clouded the search for differential heat resistance, thus it was decided to focus on faulty or absent SASP. The eight isolates in our study share three orthologs similar to those in other Clostridia: *ssp1, ssp2*, and *ssp3* (Raju et al., 2006; Galperin et al., 2012). These encode proteins containing all the major α/β-type SASP conserved domains, and show very little difference in protein sequence between clade I and clade II isolates, suggesting the presence of functional SASP-DNA protection in all isolates (Supplementary Figure 1). Additionally, the minor SASP—*ssp5*, H-type, and *tlp*—are also well conserved though not directly implicated in heat resistance.

The one SASP that demonstrated a unique feature was that encoded by the *ssp4* orthologous group. Previous research on the orthologous SASP in *C. perfringens* suggested that the presence of an aspartate (D), or other negatively charged or large amino acid at position 36 (Li and McClane, 2008) correlates with higher heat resistance when compared to other residues (Li et al., 2009). The clade I and II strains in this study displayed either a threonine (T) or isoleucine (I) residue at this position, respectively; and it is worth noting that *C. botulinum* A strain ATCC 3502 features an Ssp4 with an I at that position, yet still produces spores with a lower heat resistance than PA 3679. The lack of a negative charge at this position also does not appear to impede spore heat resistance for clade II (PA 3679) isolates. While a potential increase in spore heat resistance for PA 3679 with an I36D mutation is worth investigating, it would appear that the SASP-DNA protection mechanism provided by Ssp1-3 is already sufficient given its current robustness. As Setlow (2014b) has suggested, this dynamic hits a saturation point, beyond which more or better SASPs are no longer the limiting factor for higher spore heat resistance and the potential effect, if any, of T36I or T36D substitutions in Ssp4 for improving heat resistance of clade I isolates is unclear.

### Conserved sporulation genes

Possessing six D-alanyl-D-alanine carboxypeptidases appears typical for many *Clostridium* and *Bacillus* species. All eight isolates in this study have potential orthologs for DacB and DacF, the two carboxypeptidases which have a demonstrated effect on spore heat resistance. Dac2 through Dac5 contained all the expected conserved domains. Based on sequence homology, Dac4 is most likely the DacF ortholog, and the DacB homolog is likely either Dac2 or Dac5 (both showed similar *e*-values). Ultimately, the determination of the role of each Dac will require future experiments to determine which one is regulated by σ^F^ (DacF, expressed in the forespore) and which one by σ^E^ (DacB, expressed in the mother cell). From this study, none of the potential orthologs appeared to be significantly different between the clade I and clade II isolates (Supplementary Figure 1), thus they are likely not responsible for the differential heat resistance.

DPA synthesis in these *C. sporogenes* isolates is not controlled via a *spoVF* mechanism, though potentially is synthesized via an electron transport flavoprotein α-subunit as seen in *C. perfringens* (Orsburn et al., 2010). The EtfA_3 orthologous group lacked a C-terminal Prosite conserved domain, and EtfA_2 orthologous group had an extraneous N-terminal FerB domain, making them both unlikely candidates. The EtfA_1 product, which contained all the expected domains and had the highest sequence homology, is the most likely ortholog. Future experiments will need to replicate the experiments from Orsburn et al. (2010) in order to prove conclusively that the product of *etfA_1* is capable of DPA synthesis *in vitro* and *in vivo*. An electron transport flavoprotein is common and this phenomenon is fairly unique. Regardless, the three potential orthologous groups show a high degree of sequence conservation in all eight isolates, thus none of them are likely to account for the heat resistance difference we see between clade I and clade II isolates.

The SpmA and SpmB orthologs were present and highly conserved, generating little ambiguity about their identities. All expected domains were present, and minimal variation between clade I and clade II sequences make it unlikely that they contribute to the differential heat resistance. All additional genes examined showed a very high sequence similarity to unique orthologous groups containing representatives from all eight isolates. Plus, their involvement in spore heat resistance is mostly tangential, again making them unlikely factors in the observed change (For more information see Supplementary Table 1 and Supplementary Figure 1).

### Interrelatedness of group I *Clostridium* species and origins of the *spoVA2* locus

The phylogenetic tree produced in this study (Figure 3) was consistent with previous studies (Kenri et al., 2014; Weigand et al., 2015; Williamson et al., 2016). However, PA 3679 strains did not group with other *C. sporogenes* strains, instead clustering deep in the left branch. The Mash pairwise distances corroborated the phylogenetic tree, demonstrating not only the position of PA 3679 strains with a group of *C. botulinum*, but heterogeneity among *C. sporogenes* strains in general. Considering how very different PA 3679 strains appear from the other *C. sporogenes* strains, the assertion that *C. sporogenes* strains in general are suitable non-toxic surrogates is questionable. Most of the *C. sporogenes* strains examined lack the *spoVA2* locus, and given their phylogenetic relatedness to the clade I isolates from this study, it is likely that they possess similar low heat resistance profiles. *C. sporogenes* CDC24533 is the exception—possessing *spoVA2*—and has the potential to produce spores that are resistant to high temperatures similar to PA 3679, warranting further investigation.

The high degree of conservation observed between the *spoVA2* operons present in species from the left side of the tree (Figure 3) argues in favor of a common origin, but it is unclear if this distribution results from multiple independent acquisition events from similar sources or rather from a single acquisition in their shared common ancestor followed by independent losses. While heat resistance confers an obvious advantage to species exposed to extreme temperatures like those involved in canning processes, those conditions are rarely met in the environment and one can envision that the added benefit may be rather minimal in normal circumstances, and thus commonly lost during pruning processes. In any case, the presence of the *spoVA2* operon in botulinum neurotoxin-containing *Clostridium* species strongly argues in favor of maintaining stringent canning processes that meet or exceed spore destruction targets of heat-resistant *C. sporogenes* isolates.

## Conclusions

The high heat resistance of *Clostridium sporogenes* PA 3679 is unique among observed *C. sporogenes* strains. While this resistance is most likely influenced by the presence of an extra *spoVA2* operon, other factors including differential expression, altered function of canonical sporulation proteins and/or additional novel sporulation proteins could be involved. Further, studies will be required to circumscribe the full set of factors that confer to PA 3679 this thermal endurance and to better define the mechanisms that are involved in its endospore survival. Furthermore, because the potential for higher heat resistance also exists in both harmless and pathogenic species, strategies to detect and reduce thermal stability in foodborne organisms as well as to how maintain safe standards of food processing will need to be revisited.

## Author contributions

RB, JP, KS, and YW designed the study and drafted the manuscript. RB conducted the work and RB and JP conducted the analysis.

## Funding

This work was supported by a C.V. Starr fellowship to RB and by funds from the Illinois Institute of Technology to JP and YW was supported by an appointment to the Research Participation Program at the Center for Food Safety and Applied Nutrition administered by the Oak Ridge Institute for Science and Education via an interagency agreement between the U.S. Department of Energy and the FDA.

### Conflict of interest statement

The authors declare that the research was conducted in the absence of any commercial or financial relationships that could be construed as a potential conflict of interest.

## References

[B1] Altschul S. F. Gish W. Miller W. Myers E. W. Lipman D. J. (1990). Basic local alignment search tool. J. Mol. Biol. 215, 403–410. 10.1016/S0022-2836(05)80360-2 2231712

[B2] Bach M. L. Gilvarg C. (1966). Biosynthesis of dipicolinic acid in sporulating *Bacillus megaterium*. J. Biol. Chem. 241, 4563–4564. 4958818

[B3] Berendsen E. M. Boekhorst J. Kuipers O. P. Wells-Bennik M. H. (2016). A mobile genetic element profoundly increases heat resistance of bacterial spores. ISME J. 10, 2633–2642. 10.1038/ismej.2016.59 27105070 PMC5113849

[B4] Brown J. L. Tran-Dinh N. Chapman B. (2012). *Clostridium sporogenes* PA 3679 and its uses in the derivation of thermal processing schedules for low-acid shelf-stable foods and as a research model for proteolytic *Clostridium botulinum*. J. Food Prot. 75, 779–792. 10.4315/0362-028X.JFP-11-391 22488072

[B5] Brunt J. van Vliet A. H. van den Bos F. Carter A. T. Peck M. W. (2016). Diversity of the germination apparatus in *Clostridium botulinum* groups I, II, III, and IV. Front. Microbiol. 7:1702. 10.3389/fmicb.2016.01702 27840626 PMC5083711

[B6] Bull M. K. Olivier S. A. van Diepenbeek R. J. Kormelink F. Chapman B. (2009). Synergistic inactivation of spores of proteolytic *Clostridium botulinum* strains by high pressure and heat is strain and product dependent. Appl. Environ. Microbiol. 75, 434–445. 10.1128/AEM.01426-08 19011055 PMC2620695

[B7] Cabrera-Hernandez A. Sanchez-Salas J.-L. Paidhungat M. Setlow P. (1999). Regulation of four genes encoding small, acid-soluble spore proteins in *Bacillus subtilis*. Gene 232, 1–10. 10.1016/S0378-1119(99)00124-9 10333516

[B8] Cabrera-Martinez R. M. Setlow P. (1991). Cloning and nucleotide sequence of three genes coding for small, acid-soluble proteins of *Clostridium perfringens* spores. FEMS Microbiol. Lett. 61, 127–131. 10.1111/j.1574-6968.1991.tb04335.x 2037223

[B9] Collins M. D. East A. K. (1998). Phylogeny and taxonomy of the food-borne pathogen *Clostridium botulinum* and its neurotoxins. J. Appl. Microbiol. 84, 5–17. 10.1046/j.1365-2672.1997.00313.x 15244052

[B10] Criscuolo A. Gribaldo S. (2010). BMGE (Block Mapping and Gathering with Entropy): a new software for selection of phylogenetic informative regions from multiple sequence alignments. BMC Evol. Biol. 10:210. 10.1186/1471-2148-10-210 20626897 PMC3017758

[B11] Csárdi G. Nepusz T. (2006). The igraph software package for complex network research. Inter J. Complex Syst. 1695, 1–9. Available online at: http://interjournal.org/manuscript_abstract.php?361100992

[B12] Daniel R. A. Errington J. (1993). Cloning, DNA sequence, functional analysis and transcriptional regulation of the genes encoding dipicolinic acid synthetase required for sporulation in *Bacillus subtilis*. J. Mol. Biol. 232, 468–483. 10.1006/jmbi.1993.1403 8345520

[B13] Diao M. M. André S. Membré J.-M. (2014). Meta-analysis of D-values of proteolytic *Clostridium botulinum* and its surrogate strain Clostridium sporogenes PA 3679. Int. J. Food Microbiol. 174, 23–30. 10.1016/j.ijfoodmicro.2013.12.029 24448274

[B14] Dodds K. L. Hauschild A. H. (1989). Distribution of *Clostridium botulinum* in the environment and its significance in relation to botulism, in Proceedings of the 5th International Symposium on Microbial Ecology (Kyoto: International Society for Microbial Ecology), 472.

[B15] Donnelly M. L. Fimlaid K. A. Shen A. (2016). Characterization of *Clostridium difficile* spores lacking either *SpoVA* or dipicolinic acid synthetase. J. Bacteriol. 198, 1694–1707. 10.1128/JB.00986-15 27044622 PMC4959285

[B16] Dürre P. (2005). Sporulation in clostridia (Genetics), in Handbook on Clostridia, ed Dürre P. (Boca Raton, FL: CRC Press), 659–669.

[B17] Eichenberger P. Jensen S. T. Conlon E. M. van Ooij C. Silvaggi J. González-Pastor J.-E.. (2003). The σ^E^ regulon and the identification of additional sporulation genes in *Bacillus subtilis*. J. Mol. Biol. 327, 945–972. 10.1016/S0022-2836(03)00205-5 12662922

[B18] Emms D. M. Kelly S. (2015). OrthoFinder: solving fundamental biases in whole genome comparisons dramatically improves orthogroup inference accuracy. Genome Biol. 16:157. 10.1186/s13059-015-0721-2 26243257 PMC4531804

[B19] Galperin M. Y. Mekhedov S. L. Puigbo P. Smirnov S. Wolf Y. I. Rigden D. J. (2012). Genomic determinants of sporulation in Bacilli and Clostridia: towards the minimal set of sporulation-specific genes. Environ. Microbiol. 14, 2870–2890. 10.1111/j.1462-2920.2012.02841.x 22882546 PMC3533761

[B20] Granger A. C. Gaidamakova E. K. Matrosova V. Y. Daly M. J. Setlow P. (2011). Effects of Mn and Fe levels on *Bacillus subtilis* spore resistance and effects of Mn2+, other divalent cations, orthophosphate, and dipicolinic acid on protein resistance to ionizing radiation. Appl. Environ. Microbiol. 77, 32–40. 10.1128/AEM.01965-10 21057011 PMC3019732

[B21] Gross C. E. Vinton C. Stumbo C. R. (1946). Bacteriological studies relating to thermal processing of canned meats. V. Characteristics of putrefactive anaerobe used in thermal resistance studies. J. Food Sci. 11, 405–410. 10.1111/j.1365-2621.1946.tb16368.x 20275418

[B22] Guindon S. Dufayard J. F. Lefort V. Anisimova M. Hordijk W. Gascuel O. (2010). New algorithms and methods to estimate maximum-likelihood phylogenies: assessing the performance of PhyML 3.0. Syst. Biol. 59, 307–321. 10.1093/sysbio/syq010 20525638

[B23] Huang I. H. Waters M. Grau R. R. Sarker M. R. (2004). Disruption of the gene (*spo0A*) encoding sporulation transcription factor blocks endospore formation and enterotoxin production in enterotoxigenic *Clostridium perfringens* type A. FEMS Microbiol. Lett. 233, 233–240. 10.1111/j.1574-6968.2004.tb09487.x 15063491

[B24] Ingram M. Robinson R. H. M. (1951). A discussion of the literature on botulism in relation to acid foods. Proc. Soc. Appl. Bacteriol. 14, 73–84. 10.1111/j.1365-2672.1951.tb01995.x

[B25] Jones P. Binns D. Chang H.-Y. Fraser M. Li W. McAnulla C.. (2014). InterProScan 5: genome-scale protein function classification. Bioinformatics 30, 1236–1240. 10.1093/bioinformatics/btu031 24451626 PMC3998142

[B26] Källberg M. Wang H. Wang S. Peng J. Wang Z. Lu H.. (2012). Template-based protein structure modeling using the RaptorX web server. Nat. Protoc. 7, 1511–1522. 10.1038/nprot.2012.085 22814390 PMC4730388

[B27] Kearse M. Moir R. Wilson A. Stones-Havas S. Cheung M. Sturrock S.. (2012). Geneious Basic: an integrated and extendable desktop software platform for the organization and analysis of sequence data. Bioinformatics 28, 1647–1649. 10.1093/bioinformatics/bts199 22543367 PMC3371832

[B28] Kenri T. Sekizuka T. Yamamoto A. Iwaki M. Komiya T. Hatakeyama T.. (2014). Genetic characterization and comparison of *Clostridium botulinum* isolates from botulism cases in Japan between 2006 and 2011. Appl. Environ. Microbiol. 80, 6954–6964. 10.1128/AEM.02134-14 25192986 PMC4249013

[B29] Lee K. S. Bumbaca D. Kosman J. Setlow P. Jedrzejas M. J. (2008). Structure of a protein-DNA complex essential for DNA protection in spores of *Bacillus* species. Proc. Natl. Acad. Sci. U.S.A. 105, 2806–2811. 10.1073/pnas.0708244105 18287075 PMC2268541

[B30] Li J. McClane B. A. (2008). A novel small acid soluble protein variant is important for spore resistance of most *Clostridium perfringens* food poisoning isolates. PLoS Pathog. 4:e1000056. 10.1371/journal.ppat.1000056 18451983 PMC2323104

[B31] Li J. Paredes-Sabja D. Sarker M. R. McClane B. A. (2009). Further characterization of *Clostridium perfringens* small acid soluble protein-4 (Ssp4) properties and expression. PLoS ONE 4:e6249. 10.1371/journal.pone.0006249 19609432 PMC2706996

[B32] Li Y. Davis A. Korza G. Zhang P. Setlow B. Setlow P.. (2012). Role of a SpoVA protein in dipicolinic acid uptake into developing spores of *Bacillus subtilis*. J. Bacteriol. 194, 1875–1884. 10.1128/JB.00062-12 22328679 PMC3318455

[B33] McClung L. S. (1937). Studies on anaerobic bacteria X. heat stable and heat labile antigens in the botulinus and related groups of sporebearing anaerobes. J. Infect. Dis. 60, 122–128. 10.1093/infdis/60.1.122

[B34] Molle V. Fujita M. Jensen S. T. Eichenberger P. González-Pastor J. E. Liu J. S.. (2003). The Spo0A regulon of *Bacillus subtilis*. Mol. Microbiol. 50, 1683–1701. 10.1046/j.1365-2958.2003.03818.x 14651647

[B35] Nakamura S. Okado I. Nakashio S. Nishida S. (1977). *Clostridium sporogenes* isolates and their relationship to *C. botulinum* based on deoxyribonucleic acid reassociation. J. Gen. Microbiol. 100, 395–401. 10.1099/00221287-100-2-395 330814

[B36] Ondov B. D. Treangen T. J. Melsted P. Mallonee A. B. Bergman N. H. Koren S.. (2016). Mash: fast genome and metagenome distance estimation using MinHash. Genome Biol. 17:132. 10.1186/s13059-016-0997-x 27323842 PMC4915045

[B37] Onyenwoke R. U. Brill J. A. Farahi K. Wiegel J. (2004). Sporulation genes in members of the low G+C Gram-type-positive phylogenetic branch (Firmicutes). Arch. Microbiol. 182, 182–192. 10.1007/s00203-004-0696-y 15340788

[B38] Orsburn B. C. Melville S. B. Popham D. L. (2010). EtfA catalyses the formation of dipicolinic acid in *Clostridium perfringens*. Mol. Microbiol. 75, 178–186. 10.1111/j.1365-2958.2009.06975.x 19968785

[B39] Orsburn B. Sucre K. Popham D. L. Melville S. B. (2009). The SpmA/B and DacF proteins of *Clostridium perfringens* play important roles in spore heat resistance. FEMS Microbiol. Lett. 291, 188–194. 10.1111/j.1574-6968.2008.01454.x 19189487

[B40] Page A. J. Cummins C. A. Hunt M. Wong V. K. Reuter S. Holden M. T.. (2015). Roary: rapid large-scale prokaryote pan genome analysis. Bioinformatics 31, 3691–3693. 10.1093/bioinformatics/btv421 26198102 PMC4817141

[B41] Paidhungat M. Setlow B. Driks A. Setlow P. (2000). Characterization of spores of *Bacillus subtilis* which lack dipicolinic acid. J. Bacteriol. 182, 5505–5512. 10.1128/JB.182.19.5505-5512.2000 10986255 PMC110995

[B42] Paredes-Sabja D. Sarker N. Setlow B. Setlow P. Sarker M. R. (2008a). Roles of DacB and Spm proteins in *Clostridium perfringens* spore resistance to moist heat, chemicals, and UV radiation. Appl. Environ. Microbiol. 74, 3730–3738. 10.1128/AEM.00169-08 18441110 PMC2446547

[B43] Paredes-Sabja D. Setlow B. Setlow P. Sarker M. R. (2008b). Characterization of *Clostridium perfringens* spores that lack SpoVA proteins and dipicolinic acid. J. Bacteriol. 190, 4648–4659. 10.1128/JB.00325-08 18469104 PMC2446781

[B44] Perez-Valdespino A. Li Y. Setlow B. Ghosh S. Pan D. Korza G.. (2014). Function of the SpoVAEa and SpoVAF proteins of *Bacillus subtilis* spores. J. Bacteriol. 196, 2077–2088. 10.1128/JB.01546-14 24682327 PMC4010985

[B45] Popham D. L. Gilmore M. E. Setlow P. (1999). Roles of low-molecular-weight penicillin-binding proteins in *Bacillus subtilis* spore peptidoglycan synthesis and spore properties. J. Bacteriol. 181, 126–32. 9864321 10.1128/jb.181.1.126-132.1999 PMC103540

[B46] Ragkousi K. Eichenberger P. van Ooij C. Setlow P. (2003). Identification of a new gene essential for germination of *Bacillus subtilis* spores with Ca2+-dipicolinate. J. Bacteriol. 185, 2315–2329. 10.1128/JB.185.7.2315-2329.2003 12644503 PMC151495

[B47] Raju D. Waters M. Setlow P. Sarker M. R. (2006). Investigating the role of small, acid-soluble spore proteins (SASPs) in the resistance of *Clostridium perfringens* spores to heat. BMC Microbiol. 6:50. 10.1186/1471-2180-6-50 16759397 PMC1501028

[B48] R Core Team T (2016). R: A Language and Environment for Statistical Computing. Vienna: R Foundation for Statistical Computing.

[B49] Rossetto O. Pirazzini M. Montecucco C. (2014). Botulinum neurotoxins: genetic, structural and mechanistic insights. Nat. Rev. Microbiol. 12, 535–549. 10.1038/nrmicro3295 24975322

[B50] Schill K. M. Wang Y. Butler R. R. III. Pombert J.-F. Reddy N. R. Skinner G. E.. (2016). Genetic diversity of *Clostridium sporogenes* PA 3679 isolates obtained from different sources as resolved by pulsed-field gel electrophoresis and high-throughput sequencing. Appl. Environ. Microbiol. 82, 384–393. 10.1128/AEM.02616-15 26519392 PMC4702626

[B51] Setlow P. (2006). Spores of *Bacillus subtilis*: their resistance to and killing by radiation, heat and chemicals. J. Appl. Microbiol. 101, 514–525. 10.1111/j.1365-2672.2005.02736.x 16907802

[B52] Setlow P. (2007). I will survive: DNA protection in bacterial spores. Trends Microbiol. 15, 172–180. 10.1016/j.tim.2007.02.004 17336071

[B53] Setlow P. (2014a). Germination of spores of *Bacillus* Species: what we know and do not know. J. Bacteriol. 196, 1297–1305. 10.1128/JB.01455-13 24488313 PMC3993344

[B54] Setlow P. (2014b). Spore Resistance Properties. Microbiol. Spectr. 2, 201–215. 10.1128/microbiolspec.TBS-0003-2012 26104355

[B55] Stumbo C. R. Purohit K. S. Ramakrishnan T. V. (1975). Thermal process lethality guide for low-acid foods in metal containers. J. Food Sci. 40, 1316–1323. 10.1111/j.1365-2621.1975.tb01080.x

[B56] Sullivan M. J. Petty N. K. Beatson S. A. (2011). Easyfig: a genome comparison visualizer. Bioinformatics 27, 1009–1010. 10.1093/bioinformatics/btr039 21278367 PMC3065679

[B57] Tovar-Rojo F. Chander M. Setlow B. Setlow P. (2002). The products of the *spoVA* operon are involved in dipicolinic acid uptake into developing spores of *Bacillus subtilis*. J. Bacteriol. 184, 584–587. 10.1128/JB.184.2.584-587.2002 11751839 PMC139579

[B58] Townsend C. T. Esty J. K. Baselt F. C. (1938). Heat-resistance studies on spores of putrefactive anaerobes in relation to determination of safe processes for canned foods. J. Food Sci. 3, 323–346. 10.1111/j.1365-2621.1938.tb17065.x

[B59] Velásquez J. Schuurman-Wolters G. Birkner J. P. Abee T. Poolman B. (2014). *Bacillus subtilis* spore protein SpoVAC functions as a mechanosensitive channel. Mol. Microbiol. 92, 813–823. 10.1111/mmi.12591 24666282

[B60] Wang S. T. Setlow B. Conlon E. M. Lyon J. L. Imamura D. Sato T.. (2006). The forespore line of gene expression in *Bacillus subtilis*. J. Mol. Biol. 358, 16–37. 10.1016/j.jmb.2006.01.059 16497325

[B61] Weigand M. R. Pena-Gonzalez A. Shirey T. B. Broeker R. G. Ishaq M. K. Konstantinidis K. T.. (2015). Implications of genome-based discrimination between *Clostridium botulinum* group I and *Clostridium sporogenes* strains for bacterial taxonomy. Appl. Environ. Microbiol. 81, 5420–5429. 10.1128/AEM.01159-15 26048939 PMC4510194

[B62] Wetzel D. Fischer R.-J. (2015). Small acid-soluble spore proteins of *Clostridium acetobutylicum* are able to protect DNA *in vitro* and are specifically cleaved by germination protease GPR and spore protease YyaC. Microbiology 161, 2098–2109. 10.1099/mic.0.000162 26362088

[B63] Williamson C. H. Sahl J. W. Smith T. J. Xie G. Foley B. T. Smith L. A.. (2016). Comparative genomic analyses reveal broad diversity in botulinum-toxin-producing Clostridia. BMC Genomics 17:180. 10.1186/s12864-016-2502-z 26939550 PMC4778365

